# New Virus Variant Detection Based on the Optimal Natural Metric

**DOI:** 10.3390/genes15070891

**Published:** 2024-07-07

**Authors:** Hongyu Yu, Stephen S.-T. Yau

**Affiliations:** 1 Department of Mathematical Sciences, Tsinghua University, Beijing 100084, China; yuhy22@mails.tsinghua.edu.cn; 2 Beijing Institute of Mathematical Sciences and Applications (Bimsa), Beijing 101408, China

**Keywords:** new virus detection, optimal metric, natural vectors, SARS-CoV-2

## Abstract

The highly variable SARS-CoV-2 virus responsible for the COVID-19 pandemic frequently undergoes mutations, leading to the emergence of new variants that present novel threats to public health. The determination of these variants often relies on manual definition based on local sequence characteristics, resulting in delays in their detection relative to their actual emergence. In this study, we propose an algorithm for the automatic identification of novel variants. By leveraging the optimal natural metric for viruses based on an alignment-free perspective to measure distances between sequences, we devise a hypothesis testing framework to determine whether a given viral sequence belongs to a novel variant. Our method demonstrates high accuracy, achieving nearly 100% precision in identifying new variants of SARS-CoV-2 and HIV-1 as well as in detecting novel genera in Orthocoronavirinae. This approach holds promise for timely surveillance and management of emerging viral threats in the field of public health.

## 1. Introduction

Viruses are microorganisms that depend on host cells for replication, and are characterized by their high variability [1]. This trait is especially evident in RNA viruses such as Severe Acute Respiratory Syndrome Coronavirus-2 (SARS-CoV-2) and Human Immunodeficiency Virus-1 (HIV-1) [2,3]. The rapid mutation rates of these viruses frequently lead to the emergence of new variants with distinct pathogenicity or transmissibility, posing significant challenges to public health efforts, as evidenced by the COVID-19 pandemic [4].

Therefore, the timely identification of new variants has become a crucial issue in biology. However, current processes for discovering and defining new variants often require manual intervention. After a new sequence is obtained, it is aligned with known sequences at biologically significant regions (e.g., segments corresponding to the spike protein) in order to identify mutation sites [5]. Subsequently, official organizations such as the World Health Organization (WHO) define variants based on key mutations [6,7]. For example, the Omicron variant of SARS-CoV-2 is characterized by a series of key mutations such as K417N, T478K, and N501Y [8]. This system proved vital during the COVID-19 pandemic; however, developing a more automated variant identification system remains a valuable goal.

Our theoretical framework differs from the aforementioned approaches. It is rooted in alignment-free principles, meaning that manual identification of important segments is not required and that there is no need to spend time on sequence alignment in order to pinpoint mutations. Instead, we assess whether a new sequence differs from known variants by analyzing its statistical characteristics. As sequencing technologies advance, the pool of known sequences is expanding, amplifying the importance of more efficient alignment-free methods [9,10,11].

Among the current alignment-free methods, the natural vector approach stands out. This approach utilizes statistical moments to transform sequences into feature vectors based on different k-mers and moment orders, and has garnered widespread attention [12,13,14]. It has demonstrated significant efficacy across such diverse fields as sequence classification, phylogenetic analysis, and chromosome fusion detection [15,16,17]. The optimal natural metric, an advancement of the natural vector method, fine tunes the integration of statistical information from various k-mers and moment orders [18]. It identifies the optimal combination of features through training on real data, thereby naturally defining dissimilarities between sequences. However, no previous applications of these metrics have addressed the discovery of new categories that do not belong to the existing classification system.

In this paper, we employ the optimal natural metric to measure the distances between sequences, then extend the metric to effectively assess the distances between individual sequences and known variants. We observe distinct distributional characteristics in the distances from sequences to their non-affiliated variants, allowing us to determine whether a sequence belongs to a given variant and if it originates from an unknown new variant through hypothesis testing. We validate this method on two significant viruses, SARS-CoV-2 and HIV-1, and further analyze different genera within the Orthocoronavirinae family at a higher classification level. These numerical experiments show very promising results, with minimal Type I and Type II errors, confirming the effectiveness of our method. This approach offers significant potential for the timely detection and management of emerging viral threats in public health.

## 2. Materials and Methods

### 2.1. Materials

We utilized three types of datasets in this study. For all datasets, sequences containing ambiguous letters and sequences with unidentified categories were removed. All sequences along with their information are available in the Github repository https://github.com/BobYHY/NewVariant (accessed on 29 June 2024).

SARS-CoV-2 Dataset: The first dataset was from GISAID (https://gisaid.org accessed on 30 June 2022), encompassing SARS-CoV-2 sequences up to 30 June 2022. We included sequences categorized as variants of concern (VOC) and variants of interest (VOI). Due to the imbalance in the number of sequences for different variants (e.g., 99,955 sequences for Omicron and only 3 for Theta), we excluded variants with fewer than ten sequences and randomly sampled 500 sequences for variants with more than 500 sequences. This resulted in a final dataset of 4917 sequences from twelve variants.

HIV-1 Dataset: The second dataset was sourced from the HIV Database (https://www.hiv.lanl.gov accessed on 8 April 2022), containing HIV-1 sequences up to 8 April 2022. Initially, we considered all non-recombinant subtype cDNA sequences available in the database and excluded subtypes with fewer than ten sequences, resulting in a dataset of 5549 sequences from six subtypes. Additionally, we included 117 recombinant viruses belonging to combinations of A+B, A+C, and B+C to further explore our method.

Orthocoronavirinae Dataset: The third dataset was sourced from NCBI (https://www.ncbi.nlm.nih.gov/ accessed on 17 November 2022), comprising Orthocoronavirinae sequences available up to 17 November 2022. Except for SARS-CoV-2, where we randomly selected 500 sequences due to the high number of available sequences, all Orthocoronavirinae sequences with lengths between 25,000 and 35,000 bases were included. This process resulted in a final dataset of 3169 sequences from four genera.

### 2.2. The Optimal Natural Metric

The optimal natural metric is used to measure the dissimilarity between sequences based on natural vectors. It leverages statistical moments to extract features from sequences, followed by training of the best weighted scheme for these features based on actual datasets [18].

The natural vector method is an alignment-free approach that transforms DNA sequences into vectors of moments [12]. Consider the sequence $S=s_{1}s_{2}\ldots s_{n}$, where $s_{i},\alpha \in \left\{A,T,C,G\right\}$; we can define

(1) $$w_{\alpha }\left(s_{i}\right)=\left\{\begin{array}{c} 1,\;s_{i}=\alpha \\ 0,\;otherwise. \end{array}\right.$$

Then, the *j*-th ordered element $D_{j}^{\alpha }$ of the natural vector can be defined as

(2) $$\left\{\begin{array}{c} D_{0}^{\alpha }=n_{\alpha }=\sum_{i=1}^{n}w_{\alpha }\left(s_{i}\right)\\ D_{1}^{\alpha }=\mu_{\alpha }=\sum_{i=1}^{n}\frac{i}{n_{\alpha }}w_{\alpha }\left(s_{i}\right)\\ D_{j}^{\alpha }=\sum_{i=1}^{n}\frac{{\left(i-\mu_{\alpha }\right)}^{j}}{n_{\alpha }^{j-1}n^{j-1}}w_{\alpha }\left(s_{i}\right)\;\left(j=2,3,4,\ldots \right), \end{array}\right.$$

where $n=n_{A}+n_{T}+n_{C}+n_{G}$. The distance between *j*-th ordered elements of two natural vectors refers to the distance between their corresponding $\left(D_{j}^{A},D_{j}^{T},D_{j}^{C},D_{j}^{G}\right)$.

The *k*-mer natural vector method extends the natural vector method [13]. A *k*-mer is a string composed of *k* nucleotides, resulting in $4^{k}$ possible *k*-mers, denoted as $l_{1},\ldots ,l_{4^{k}}$. For a sequence $S=s_{1}s_{2}\ldots s_{n}$, we can view it as a sequence consisting of n−k+1 *k*-mers, i.e., $\left(s_{1}\ldots s_{k}\right)\ldots \left(s_{n-k+1}\ldots s_{n}\right)$. We can define the *j*-th ordered moments of *k*-mers similar to those of nucleotides, and thereby define the *k*-mer natural vector (if $D_{0}^{l_{i}}=0$, then we set $D_{1}^{l_{i}}=D_{2}^{l_{i}}=\ldots =0$).

Given two DNA sequences *a* and *b*, the Euclidean distance between the *j*-th ordered elements of their *k*-mer natural vectors is defined as $dis_{kj}\left(a,b\right)$. With an arbitrarily chosen weight matrix $w_{kj}$, we can define a weighting metric as

(3) $$Dis^{w}\left(a,b\right)=\sum_{k=1}^{K}\sum_{j=0}^{J}w_{kj}dis_{kj}\left(a,b\right).$$

In this metric, different $dis_{kj}$ are used to capture differences in various types of information within sequences. By integrating them, a more comprehensive measurement between sequences can be obtained. We aim to achieve the best classification performance of the metric under a set of weights, where the performance is measured by the 1NN accuracy [19]. Specifically, we seek to ensure that sequences and their nearest neighbors belong to the same class under the chosen metric. We can smooth the 1NN accuracy and use a gradient-based algorithm to calculate the optimal weights. In applications, we set K=9 and J=2, and train these weights based on all complete virus reference sequences [18]. The specific optimal weight matrix are shown in the Table 1. In the subsequent text, we refer to the metric corresponding to these weights as *Dis.*

### 2.3. New Virus Detection Method

In the preceding section, we have defined the metric *Dis* between individual sequences. In the subsequent discussion, we extend this definition to encompass the distance from a single sequence to a set of sequences.

Considering that the known classification data of sequences may occasionally contain errors, we have devised a robust distance measure $Dis^{R}$ by computing the distance from a sequence to its *R*-th nearest neighbor in the set, which serves as the distance from the sequence to the set of sequences.

Assuming that all sequences can be divided into *M* categories, denoted as $A_{1}$,…,$A_{M}$, we can define the following sets of numbers:

(4) $$\begin{array}{cc} I_{out}\left(A_{i},A_{j}\right):= & \left\{Dis^{R}\left(a_{i},A_{j}\right)|a_{i}\in A_{i}\right\}\;\left(i\neq j\right)\\ I_{in}\left(A_{i}\right):= & \left\{Dis^{R}\left(a_{i},A_{i}\setminus \left\{a_{i}\right\}\right)|a_{i}\in A_{i}\right\}\\ I_{out}:= & \underset{i\neq j}{⋃}I_{out}\left(A_{i},A_{j}\right)\\ I_{in}:= & \overset{M}{\underset{i=1}{⋃}}I_{in}\left(A_{i}\right) \end{array}$$

where $I_{out}\left(A_{i},A_{j}\right)$ reflects the distribution of $Dis^{R}$ from sequences in $A_{i}$ to the set $A_{j}$ and its union $I_{out}$ reflects the distance characteristics when sequences are not within a certain category.

Moreover, $I_{in}\left(A_{i}\right)$ reflects the distribution of $Dis^{R}$ from sequences in $A_{i}$ to the set composed of other sequences within the same category, while its union $I_{in}$ reflects the distance characteristics when sequences are within a certain category.

In practical applications, significant differences are observed between the two sets of numbers $I_{in}$ and $I_{out}$. Specifically, they can be separated by a certain threshold *B*. Suppose that a new sequence *a* appears; if $Dis^{R}\left(a,A_{i}\right)>B$, then *a* can be considered to not belonging to $A_{i}$. If $\min_{i=1,\ldots ,M}Dis^{R}\left(a,A_{i}\right)>B$, then *a* does not belong to any known category, indicating that it belongs to a new category.

A straightforward choice for *B* is $B=\max I_{in}$; however, we aim for greater robustness of the algorithm to the data. Therefore, we consider the 99% quantile of $I_{in}$, i.e., we select *B* such that *B* is just greater than 99% of values in $I_{in}$.

The requirement for robustness is positively correlated with the amount of errors in the dataset. Therefore, for the selection of *R*, we associate it with the number of sequences in the largest category. We define $R:=1+\max_{i}\left\lfloor \frac{card(A_{i})}{1000}\right\rfloor$, where $card(A_{i})$ denotes the number of sequences in class $A_{i}$ and ⌊x⌋ means the nearest smaller integer for *x*.

**Figure 1:**
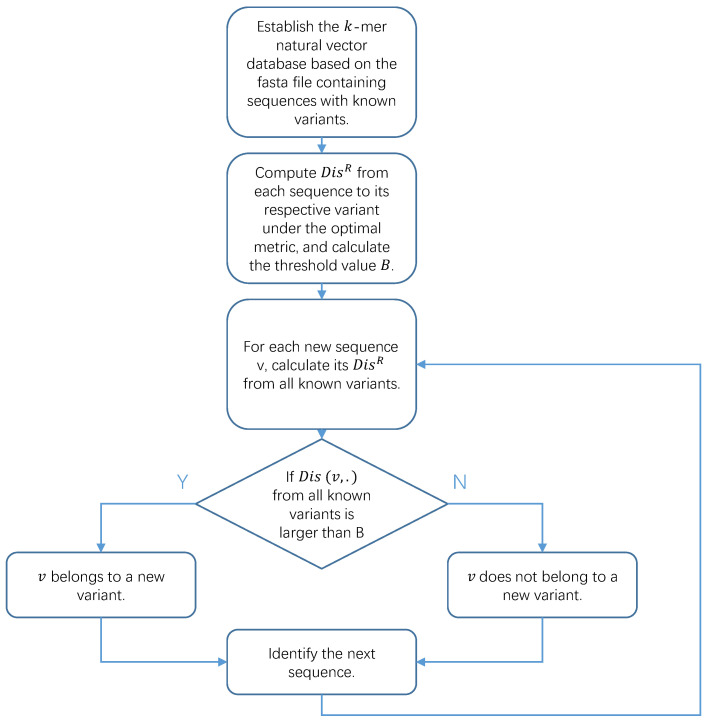
Flowchart of Algorithm 1.

We summarize our method in Algorithm 1. The first four steps can be performed in advance before the arrival of a new sequence, and these calculations need to be performed only once with multiple query sequences, ensuring high efficiency. The code can be found in both the Supplementary Materials and the Github repository https://github.com/BobYHY/NewVariant (accessed on 29 June 2024). In Figure 1, we present a flowchart to assist in understanding how we obtained our results from the original data.

We evaluate the performance of the algorithm from two perspectives. In statistical terms, hypothesis testing errors are divided into Type I errors and Type II errors [20]. Here, a Type I error refers to failing to identify sequences from a new category, while a Type II error refers to misidentifying sequences from an existing category as sequences from a new category. For a given dataset, we follow the leave-one-out approach to test the algorithm’s error rate. When assessing the Type I error rate, we examine each sequence $v_{i}$ in succession as a new sequence. Here, all sequences except those in the same category as $v_{i}$ are regarded as known sequences, and the probability of failing to detect the new virus is calculated. In evaluating the Type II error rate, we again consider each sequence in sequence as a new sequence. This time, all other known sequences, including those in the same category as $v_{i}$, are considered known, and the probability of incorrectly categorizing the sequence as belonging to a new category is computed.
**Algorithm 1** New variant detection method**Require:** Sequences from *M* known categories $A_{1},\ldots ,A_{M}$. New sequence *v*.1:Compute pairwise distances for known sequences using the optimal natural metric.2:Determine $R:=1+\max_{i}\left\lfloor \frac{card(A_{i})}{1000}\right\rfloor$.3:Compute $Dis^{R}$ for all sequences with respect to their respective categories to obtain $I_{in}$.4:Determine *B* based on the 99% quantile of $I_{in}$.5:Given a new sequence *v*, calculate $d=\min_{i=1,\ldots ,M}Dis^{R}\left(v,A_{i}\right)$.6:**if** d>B **then**7:   Claim that *v* belongs to a new category.8:**else**9:   Assert that *v* does not belong to a new category and its classification result can be obtained from its nearest neighbors.10:**end if**

## 3. Results

### 3.1. SARS-CoV-2

The most significant variants of SARS-CoV-2 belong to the category of variants of concern (VOC), which includes Alpha, Beta, Gamma, Delta, and Omicron. Additionally, there are variants of interest (VOI) that are also noteworthy. After removing variants with insufficient sequence counts, seven VOIs remain: Epsilon, Zeta, Eta, Iota, Kappa, Lambda, and Mu. These twelve variants comprise the 4917 sequences that we analyze.

To begin with, we use the optimal natural metric to classify sequences by the 1NN algorithm with the leave-one-out strategy, achieving an accuracy of 99.9%. This high accuracy demonstrates that the optimal natural metric effectively reflects the relationships between SARS-CoV-2 RNA sequences.

**Figure 2:**
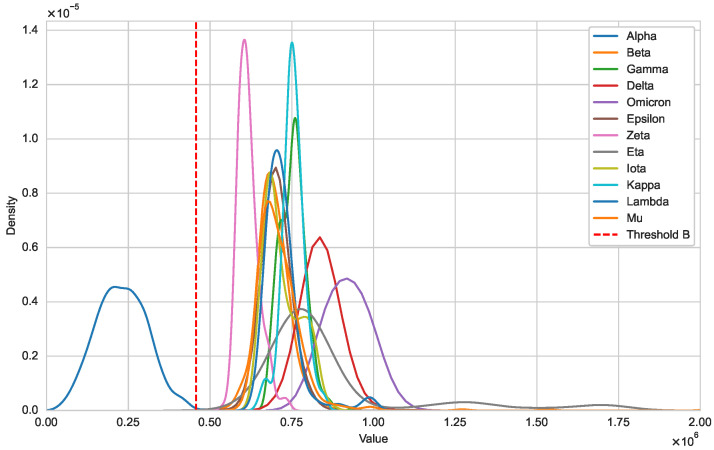
Distribution of $I_{in}\left(Alpha\right)$ and $I_{out}\left(\cdot ,Alpha\right)$ (SARS-CoV-2).

**Figure 3:**
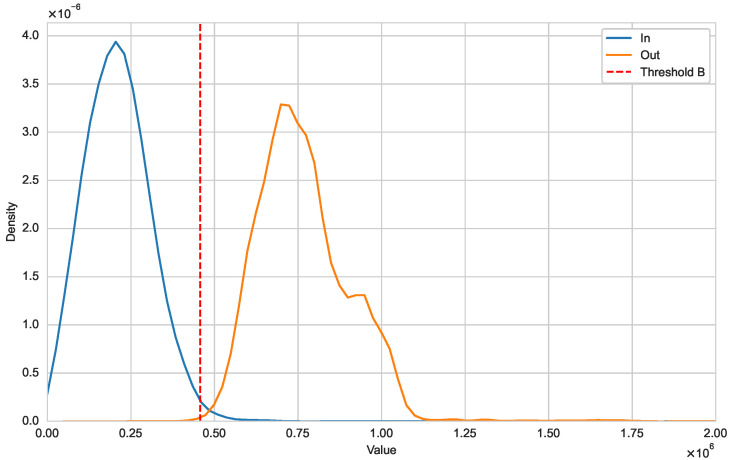
Distribution of $I_{in}$ and $I_{out}$ (SARS-CoV-2).

Next, we illustrate that the optimal natural metric is a viable approach for detecting new variants. In Figure 2, we use kernel density estimation to show the distribution differences between $I_{in}\left(Alpha\right)$ and $I_{out}\left(\cdot ,Alpha\right)$ [21]. It is evident that the distribution of $Dis^{R}\left(\cdot ,Alpha\right)$ for sequences within variant Alpha falls within the threshold, forming a unimodal distribution. In contrast, $Dis^{R}\left(\cdot ,Alpha\right)$ for sequences from other variants are outside this threshold. Figure 3 further illustrates the distributional differences between $I_{in}$ and $I_{out}$. The distinct separation between these two distributions indicates that our threshold can effectively distinguish whether a sequence belongs to a specific variant class.

We test Algorithm 1 for various SARS-CoV-2 variants using the previously described strategies for evaluating Type I and Type II errors. In the Type I error test, we assume that sequences from a given variant have never been discovered before and assess whether a newly appearing sequence from that variant can be correctly identified as new. In the Type II error test, we evaluate whether a sequence from known variants can be mistakenly identified as a new variant. As shown in Table 2, both types of errors are kept below 1%, which indicates a low rate of false positives and false negatives, yielding highly satisfactory results.

### 3.2. HIV-1

HIV-1 is categorized into a major group (M) and three minor groups (O, N, P). The major group is further subdivided into subtypes: A, B, C, D, E, F, G, H, I, J, K, L [22,23,24,25,26]. After excluding subtypes with insufficient sequence counts, six subtypes remain: A, B, C, D, F, G. These six variants comprise the 5549 sequences that we analyze.

**Figure A1:**
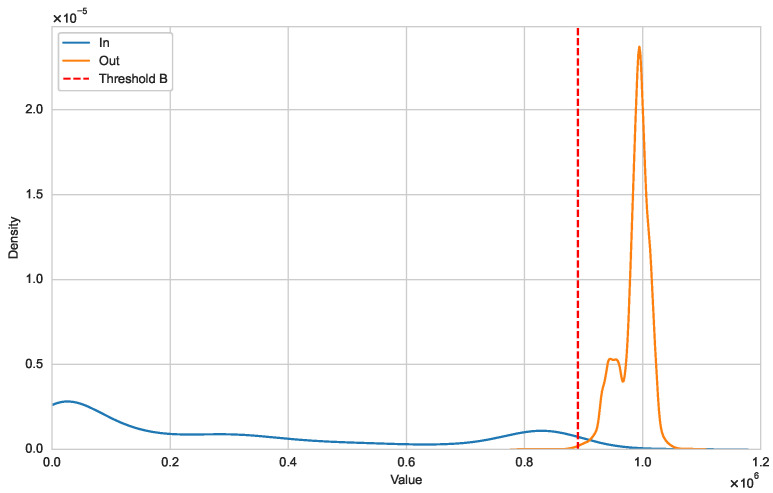
Distribution of $I_{in}$ and $I_{out}$ (HIV-1).

We repeat the aforementioned analysis for the HIV-1 virus. Initially, we determine that the 1NN classification accuracy using the optimal natural metric is 99.8%, demonstrating a remarkably high level of accuracy. In Figure A1, significant disparities can be observed between $I_{in}$ and $I_{out}$, consistent with our previous findings. Subsequently, we evaluate Algorithm 1, recording Type I and Type II errors at 0.94% and 0.87%, respectively. These results indicate promising performance of the algorithm in classifying HIV-1 variants.

One notable characteristic of HIV-1 is the significant attention given to recombinant viruses, which exhibit properties of multiple subtypes without any single subtype being dominant (if one subtype is dominant, then it is classified as pure) [27]. We aim to investigate how these recombinant viruses perform with our algorithm. Specifically, we consider three combinations: A+B, A+C, and B+C, comprising 10, 38, and 69 sequences, respectively.

Generally speaking, for recombinant viruses of the X+Y type, there are four potential identification outcomes. The first outcome is when the virus is identified as either X or Y, indicating a closer similarity to one of the recombinant parents. This suggests that the virus should be assigned to the closer category. The second outcome occurs when the virus is recognized as within both X and Y, with distances to both variants falling within the boundary. This suggests similarly short distances to both parents. The third outcome is when the virus is identified as a new variant, with distances to all known variants falling outside the boundary. This indicates significant differences between the virus and both of its parents, suggesting that it should be regarded as a new variant instead of solely a recombinant. The fourth outcome occurs when none of the above three scenarios apply, such as being classified as Z type or X+Z type. Only this fourth scenario implies an error, as it contradicts the actual situation.

Out of the 117 recombinant sequences we analyzed, only three fell into the fourth category, representing misclassifications, with the remaining sequences providing valuable biological classification suggestions. This underscores the overall effectiveness of our method. In the following parts, we illustrate the assistance that our method can provide using specific examples.

**Figure 4:**
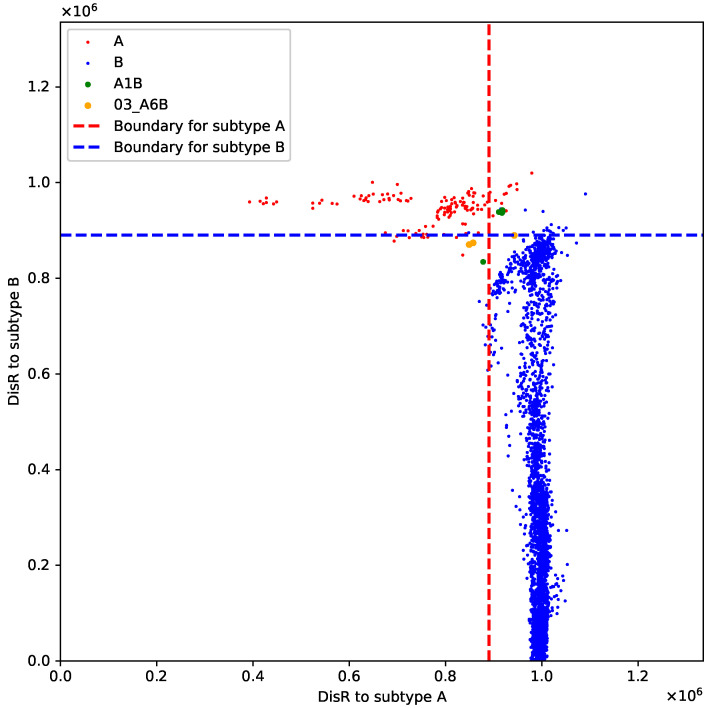
Scatter plot of $Dis^{R}$ distribution for four types of viruses: A, B, A1B, 03_A6B.

For the A+B recombinant sequences, those identified as new sequences all belong to subtype A1B, while another subtype, 03_A6B, is mostly identified as belonging to both A and B. In Figure 4, we further illustrate this result using the points $\left(Dis^{R}\left(\cdot ,A\right),Dis^{R}\left(\cdot ,B\right)\right)$ for different subtypes. This suggests that A1B is more distantly related compared to our given dataset. For the A+C recombinant sequences, A2C is consistently recognized as a new variant, whereas A1C shows inconsistent results, indicating that A2C is a more internally homogeneous subtype and is more distantly related to its parent variants. For the B+C recombinant sequences, six out of eight subtypes are consistently recognized as C, implying that these subtypes are more similar to variant C. These results can provide valuable insights into different subtypes within a recombinant group.

### 3.3. Orthocoronavirinae

Coronaviridae is a family of positive-sense single-stranded RNA viruses that cause diseases in mammals and birds [28]. They are named for their characteristic crown-like appearance, attributed to the presence of spike proteins on their surface [29]. Within the Coronaviridae family, the Orthocoronavirinae subfamily represents the vast majority, comprising four genera: Alphacoronavirus, Betacoronavirus, Gammacoronavirus, and Deltacoronavirus [30].

**Figure A2:**
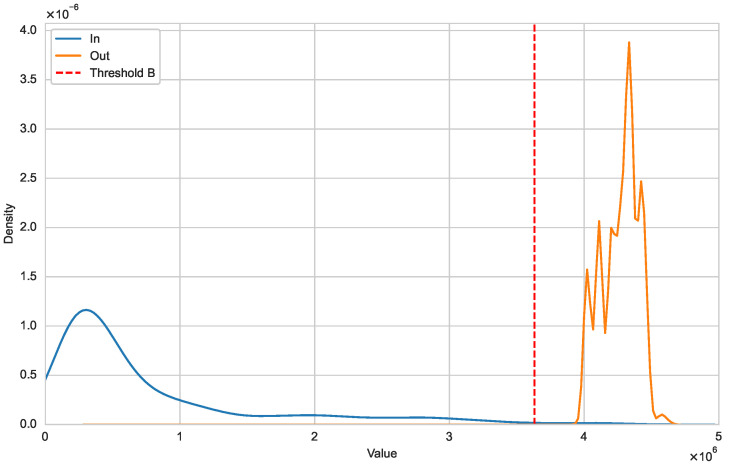
Distribution of $I_{in}$ and $I_{out}$ (Orthocoronavirinae).

In our previous analyses, we have concentrated on detecting new variants of specific viruses. Now, we expand our focus to include the identification of new genera. Initially, we calculate the 1NN classification accuracy at the genus level using the optimal natural metric, achieving a perfect score of 99.9%. In Figure A2, it can be noted that $I_{in}$ and $I_{out}$ display even greater disparities compared to the previous analysis at the variant level. This is easily comprehensible, as the discrepancies between genera are more pronounced than those between variants.

Subsequently, we assess Algorithm 1, recording Type I and Type II errors at 0.03% and 0.98%, respectively. This outcome slightly surpasses the previous viral analyses, signifying the efficacy of our method at higher classification levels.

### 3.4. Time Complexity Analysis

Our approach incorporates an alignment-free perspective, significantly reducing its time complexity. We assume a total of *N* sequences with known labels, each with a length of O(L), along with *M* known variants, where each variant comprises O(N/M) sequences alongside *V* new sequences with unknown labels. Given that the computational complexity of a single *k*-mer natural vector is O(L), the overall time complexity of the algorithm is $O(NL+N^{2}/M+V\left(L+N\right))$, with three terms corresponding to calculating the natural vectors, calculating the boundary, and computation upon the arrival of new sequences, respectively.

When using the alignment method, although search algorithms such as BLAST can quickly identify candidate similar sequences, calculating the actual distance necessitates algorithms with a time complexity of $O(L^{2})$ [31,32]. Moreover, the distance must be computed between every pair of sequences, while the alignment-free approach requires only a single embedding per sequence. Therefore, if the distance calculation component of our method were replaced with the alignment method, the overall algorithmic complexity would increase to $O(N^{2}L^{2}/M+VNL^{2})$, with two terms representing the boundary calculation and computation upon the arrival of new sequences, respectively. This is is much slower than alignment-free approaches.

To conduct a further comparison using a small sample, we randomly selected thirty sequences from each of the five variants within the VOC of SARS-CoV-2, compiling a dataset of 150 sequences. We then applied both the original method and the alignment-based approach to perform the same leave-one-out test as mentioned earlier. The time expenditure of the original method on a standard PC with CPU 2.3 GHz was 2 min and 15 s, and the Type I error and Type II error of the test were 2.00% and 1.33%, respectively. However, using the alignment-based method (employing the default parameters for global alignment in the Align.PairwiseAligner function from Biopython (version 1.7.9), with the additive inverse of the match score as the similarity metric) takes 12 h and 37 min, and the Type I error reaches 97%, indicating that the method is completely ineffective. It is worth noting that alignment methods offer various parameter options, and there may be certain configurations that can potentially make the method work. In terms of time efficiency alone, however, it is nearly impossible to process large datasets using this approach.

## 4. Discussion

In this paper, we have employed the optimal natural metric, which demonstrated exceptional performance across three distinct datasets, to measure the similarity between sequences. Using this metric, we have developed a hypothesis testing-based algorithm capable of quickly determining whether a sequence belongs to an unknown category. Our approach achieves nearly 100% accuracy in detecting new variants of SARS-CoV-2, new variants of HIV-1, and new genera within Orthocoronavirinae. Additionally, for HIV-1 our approach provides deeper insights into recombinant viruses by evaluating their relationships with their parental strains.

The significance of our method lies in two main aspects. First, in an era where pandemics are of increasing concern, the ability to rapidly detect new variants is crucial. Current methods for defining new variants rely heavily on manual selection, requiring the identification of key regions for alignment and specific mutations. Our method offers an automated approach to identifying new variants, reducing dependence on labor-intensive processes. In the process of infectious disease control and management, even after defining the known variants, there is always concern about the potential emergence of new threatening variants. Our method allows for the efficient detection of new variants based purely on sequence data, without the need to rely on biologists to identify critical regions of the virus. This capability is of significant importance.

Second, our method advances alignment-free techniques. Alignment-free methods which do not require time-consuming alignment processes and are not dependent on conserved regions are becoming increasingly important in the era of big data. Previous research has proposed various methods for extracting and comparing sequence features, with the optimal natural metric being one of the most effective. However, these methods typically only classify sequences when all categories are known, failing to address the presence of new categories. Our approach fills this gap by enabling the identification of sequences from previously unknown categories.

Certainly, our method has limitations that require further investigation in future studies. A primary issue is that our approach handles relatively complete sequences, and cannot determine whether a shorter unknown segment of a virus belongs to a new category. Therefore, while our method is very useful for disease control and management where complete sequences can be obtained, it is currently less effective in areas where complete sequences are not available. For example, the study of meta-viromes must often deal with a large number of mixed short sequences from different pathogens. If future research could extend our algorithm to make it effective on fragment sequences as well, this would be highly beneficial.

## Figures and Tables

**Table 1:** The optimal weight matrix for viruses.

*k*-mer/Order	0	1	2
1	$3.5\times 10^{-2}$	$3.9\times 10^{-2}$	$3.0\times 10^{-1}$
2	$3.1\times 10^{-2}$	$1.4\times 10^{-2}$	$8.5\times 10^{-2}$
3	$2.1\times 10^{-1}$	$9.7\times 10^{-3}$	$3.7\times 10^{-2}$
4	$4.9\times 10^{-1}$	$1.8\times 10^{-3}$	$2.6\times 10^{-3}$
5	$7.6\times 10^{-1}$	$1.3\times 10^{-3}$	$5.4\times 10^{-3}$
6	$1.0\times 10^{-0}$	$1.8\times 10^{-3}$	$1.6\times 10^{-3}$
7	$8.4\times 10^{-1}$	$1.1\times 10^{-3}$	$1.1\times 10^{-4}$
8	$7.1\times 10^{-1}$	$1.0\times 10^{-3}$	$9.3\times 10^{-4}$
9	$1.8\times 10^{-1}$	$8.1\times 10^{-4}$	$5.1\times 10^{-6}$

**Table 2:** Two error rates for Algorithm 1 for three datasets.

Dataset	Type I Error	Type II Error
SARS-CoV-2	0.94%	0.96%
HIV-1	0.94%	0.87%
Orthocoronavirinae	0.03%	0.98%

## Data Availability

The code and data are available in the Github repository https://github.com/BobYHY/NewVariant (accessed on 29 June 2024).

## Supplementary Materials

The following supporting information can be downloaded at: https://www.mdpi.com/article/10.3390/genes15070891/s1, Data S1: SARSCoV2.csv; Data S2: SARSCoV2.fasta; Data S3: HIV1.csv; Data S4: HIV1.fasta; Data S5: Orthocoronavirinae.csv; Data S6: Orthocoronavirinae.fasta; Code S1: metric.py; Code S2: algorithm1.py.

## Abbreviations

The following abbreviations are used in this manuscript:

COVID-19	Coronavirus Disease 2019
SARS-CoV-2	Severe Acute Respiratory Syndrome Coronavirus-2
HIV-1	Human Immunodeficiency Virus-1
WHO	World Health Organization
VOC	Variants Of Concern
VOI	Variants Of Interest
NCBI	National Center for Biotechnology Information
NN	Nearest Neighbor
